# Supplementary material for: The adaptive architecture of tRNA dependencies across physiological tumor environments
Source: bioRxiv. 2026 Oct 9:2026.10.08.757597. Preprint. [Version 1] doi: 10.64898/2026.10.08.757597 (PMC13692209; doi:10.64898/2026.10.08.757597)
Supplement: Supplementary file 1 [file NIHPP2026.10.08.757597v1-supplement-1.pdf]

bioRxiv preprint doi: https://doi.org/10.64898/2026.10.08.757597; this version posted October 9, 2026. The copyright holder for this preprint (which was not certified by peer review) is the author/funder, who has granted bioRxiv a license to display the preprint in perpetuity. It is made available under a CC-BY 4.0 International license.

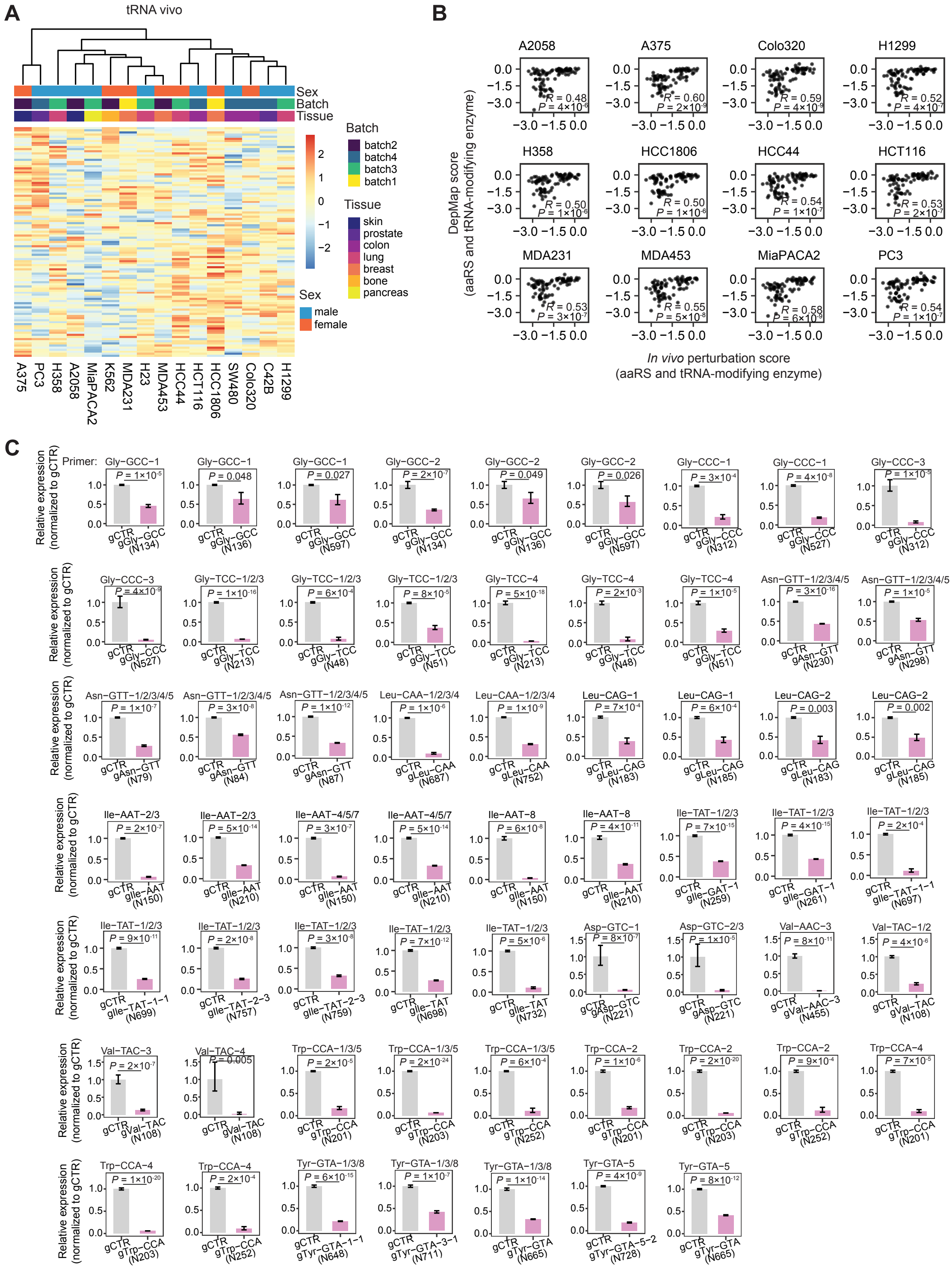

Figure S1 | Profiles of *in vivo* tRNA CRISPRi perturbations. **(A)** Heatmap showing *in vivo* tRNA CRISPRi perturbation profiles, with hierarchical clustering applied to group similar response patterns. **(B)** Scatter plot comparing the perturbation effects of aaRSs and tRNA-modifying enzymes estimated in this study with those reported in DepMap across all available cell lines. Each point represents one aaRS or tRNA-modifying enzyme. *R* and *P* values were calculated using Pearson correlation. **(C)** Bar plots showing the relative expression of target tRNA molecules upon single-guide CRISPRi-mediated tRNA knockdown in MDA-MB-231 cells compared to the non-targeting control (gCTR), quantified by RT–qPCR. Guide numbers in parentheses correspond to those used in the pooled CRISPRi screen. 5S rRNA served as the endogenous control for RT–qPCR quantification. Primers are listed. Bars represent the mean of biological and technical replicates, and error bars denote the standard error. *P* values were calculated from raw measurements using one-tailed *t* tests.

bioRxiv preprint doi: https://doi.org/10.64898/2026.10.08.757597; this version posted October 9, 2026. The copyright holder for this preprint (which was not certified by peer review) is the author/funder, who has granted bioRxiv a license to display the preprint in perpetuity. It is made available under a CC-BY 4.0 International license.

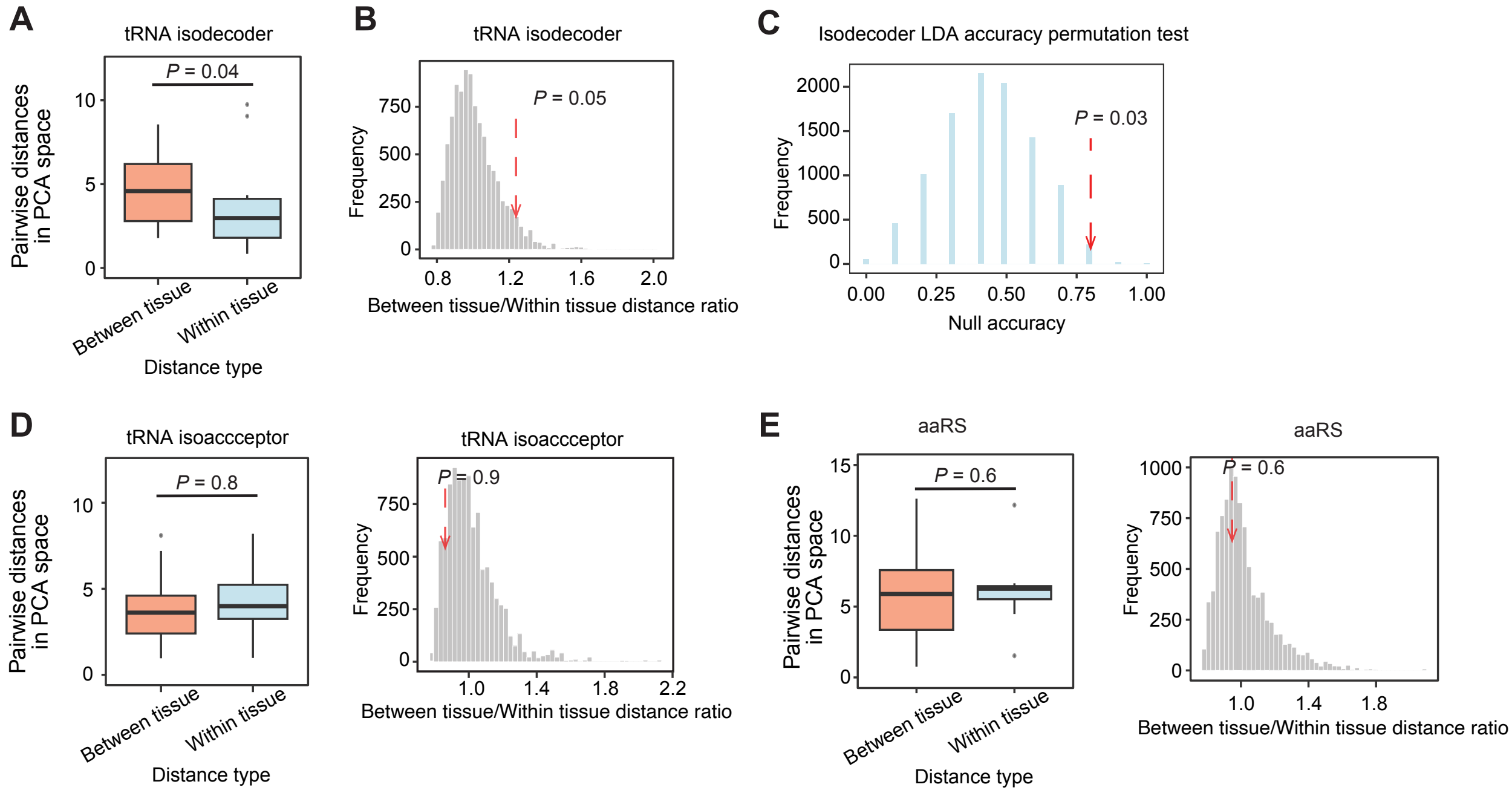

Figure S2 | Analyses of tissue-specific CRISPRi perturbation profiles of tRNA isodecoders, isoacceptors, aaRSs, and tRNA-modifying enzymes. (A) Boxplots showing the distribution of pair-wise within-tissue and between-tissue Euclidean distances in PCA space (from Figure 1D) for tRNA isodecoder perturbation scores. Within-tissue distances were calculated only for tissues with at least three cell lines. Boxplots represent the median (center line), interquartile range (box), and 1.5× interquartile range (whiskers); individual points indicate outliers. **(B)** Histogram showing the null distribution of the ratio between between-tissue and within-tissue Euclidean distances in PCA space (from Figure 1D) for tRNA isodecoder perturbation scores, obtained by random shuffling ($N = 10{,}000$). **(C)** Histogram showing the null distribution of LDA classifier accuracy in PCA space (from Figure 1D) for tRNA isodecoder perturbation scores, obtained by random shuffling ($N = 10{,}000$). **(D)** Similar to **(A–B)**, but performed at the tRNA isoacceptor level. **(E)** Similar to **(A–B)**, but performed for aaRS perturbation profiles.

bioRxiv preprint doi: https://doi.org/10.64898/2026.10.08.757597; this version posted October 9, 2026. The copyright holder for this preprint (which was not certified by peer review) is the author/funder, who has granted bioRxiv a license to display the preprint in perpetuity. It is made available under a CC-BY 4.0 International license.

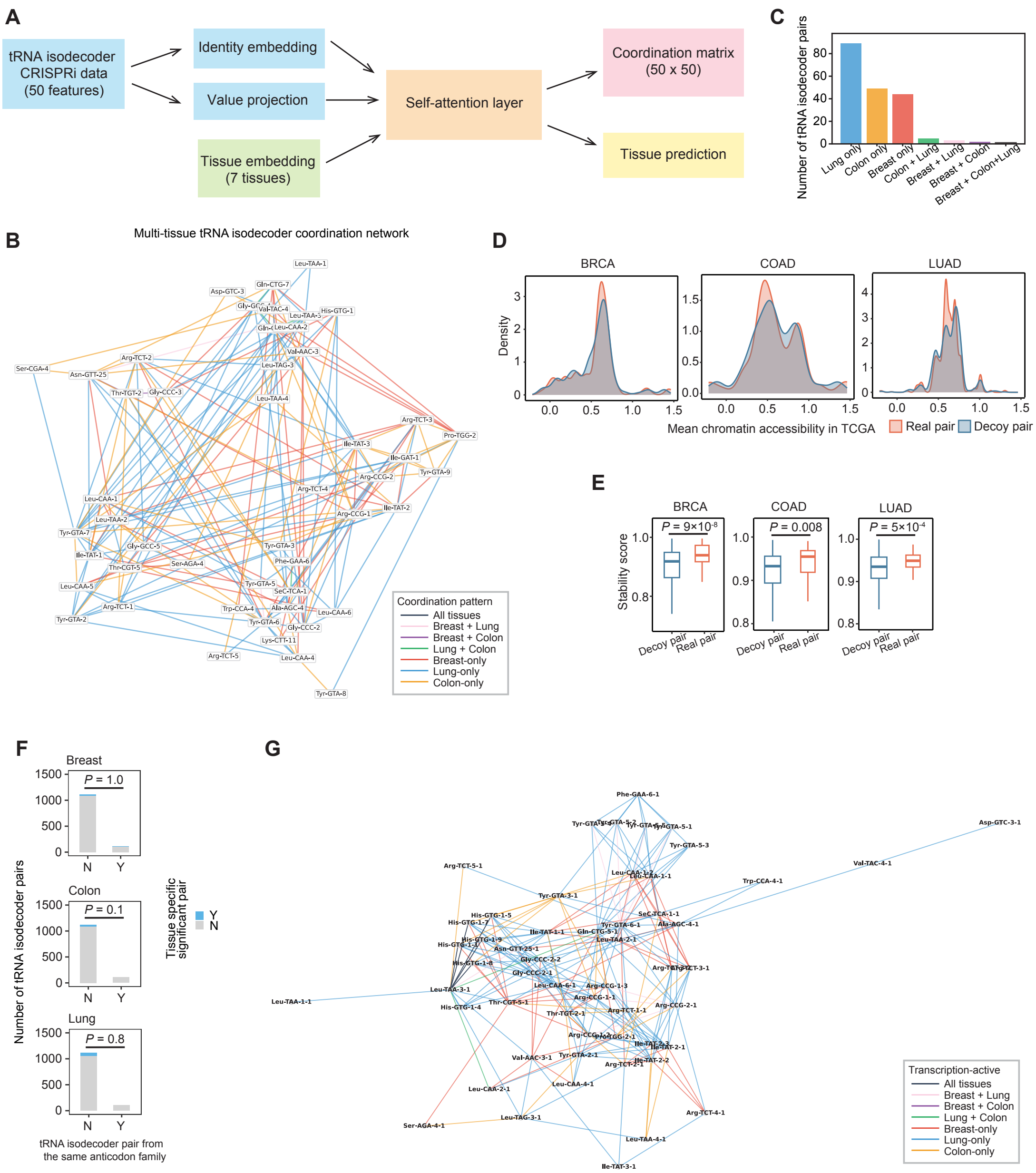

Figure S3 | Distinct structures underlie tissue-dependent tRNA isodecoder essentiality. **(A)** Schematic illustration of the multi-tissue self-attention model with contrastive learning used to identify shared and tissue-specific associative structures among tRNA isodecoders. **(B)** Network representation of significant tRNA isodecoder coordination pairs, highlighting shared and tissue-specific modules. Significant pairs were identified by permutation testing ($P < 0.05$) and stability > 0.7. **(C)** Bar plot summarizing the numbers of tissue-specific and shared significant tRNA isodecoder pairs shown in **B**. **(D)** Density plot showing the distribution of chromatin accessibility for real coordination pairs (i, j) and matched chromatin accessibility–controlled decoy partners (j') derived from TCGA data for the corresponding cancer types. **(E)** Box plot showing the stability of significant real pairs compared with chromatin-matched decoy pairs in the multi-tissue self-attention model. *P*-values were calculated using paired Mann–Whitney *U* tests. **(F)** Bar plot showing the proportion of significant tRNA isodecoder pairs arising from the same amino acid family (Y, blue) or different families (N, gray). *P*-values were calculated using Fisher's exact test. **(G)** Network representation of active tRNA loci inferred from H3K4me3 ENCODE data, corresponding to the significant tRNA isodecoder coordination pairs in **(B)**.

bioRxiv preprint doi: https://doi.org/10.64898/2026.10.08.757597; this version posted October 9, 2026. The copyright holder for this preprint (which was not certified by peer review) is the author/funder, who has granted bioRxiv a license to display the preprint in perpetuity. It is made available under a CC-BY 4.0 International license.

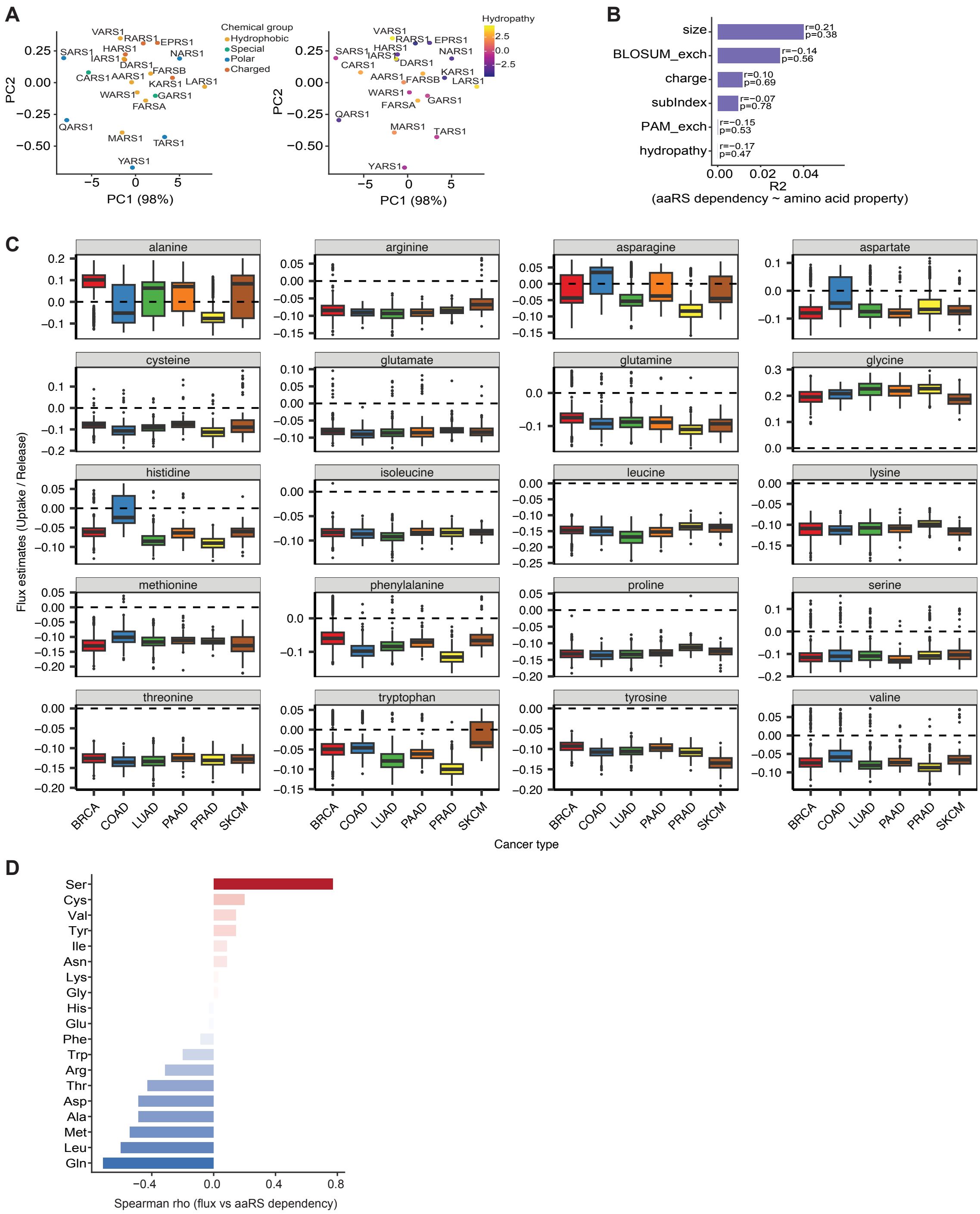

Figure S4 | aaRS dependencies in tumor physiological states. **(A)** Principal component analysis (PCA) of aminoacyl-tRNA synthetase (aaRS) dependency profiles across cancer cell lines. The same projection is colored by amino-acid chemical class (left) and hydropathy (right). **(B)** Variance in mean aaRS dependency explained (R^2) by individual amino-acid properties. BLOSUM_exch and PAM_exch represent the mean off-diagonal substitution scores derived from the BLOSUM62 and PAM250 matrices, respectively. subIndexEnc denotes the encoded amino acid substitution propensity. **(C)** Inferred amino acid metabolic flux across TCGA cancer types. Amino acid exchange fluxes were inferred from TCGA RNA-seq data. **(D)** Amino-acid-specific associations between metabolic flux and aaRS dependency. For each amino acid, Spearman correlations were calculated between median metabolic flux and mean dependency of the cognate aaRS across matched cancer types. aaRS dependency was averaged across cell lines within each TCGA-matched lineage and assigned to the amino acid charged by each synthetase before integration with metabolic flux estimates.

bioRxiv preprint doi: https://doi.org/10.64898/2026.10.08.757597; this version posted October 9, 2026. The copyright holder for this preprint (which was not certified by peer review) is the author/funder, who has granted bioRxiv a license to display the preprint in perpetuity. It is made available under a CC-BY 4.0 International license.

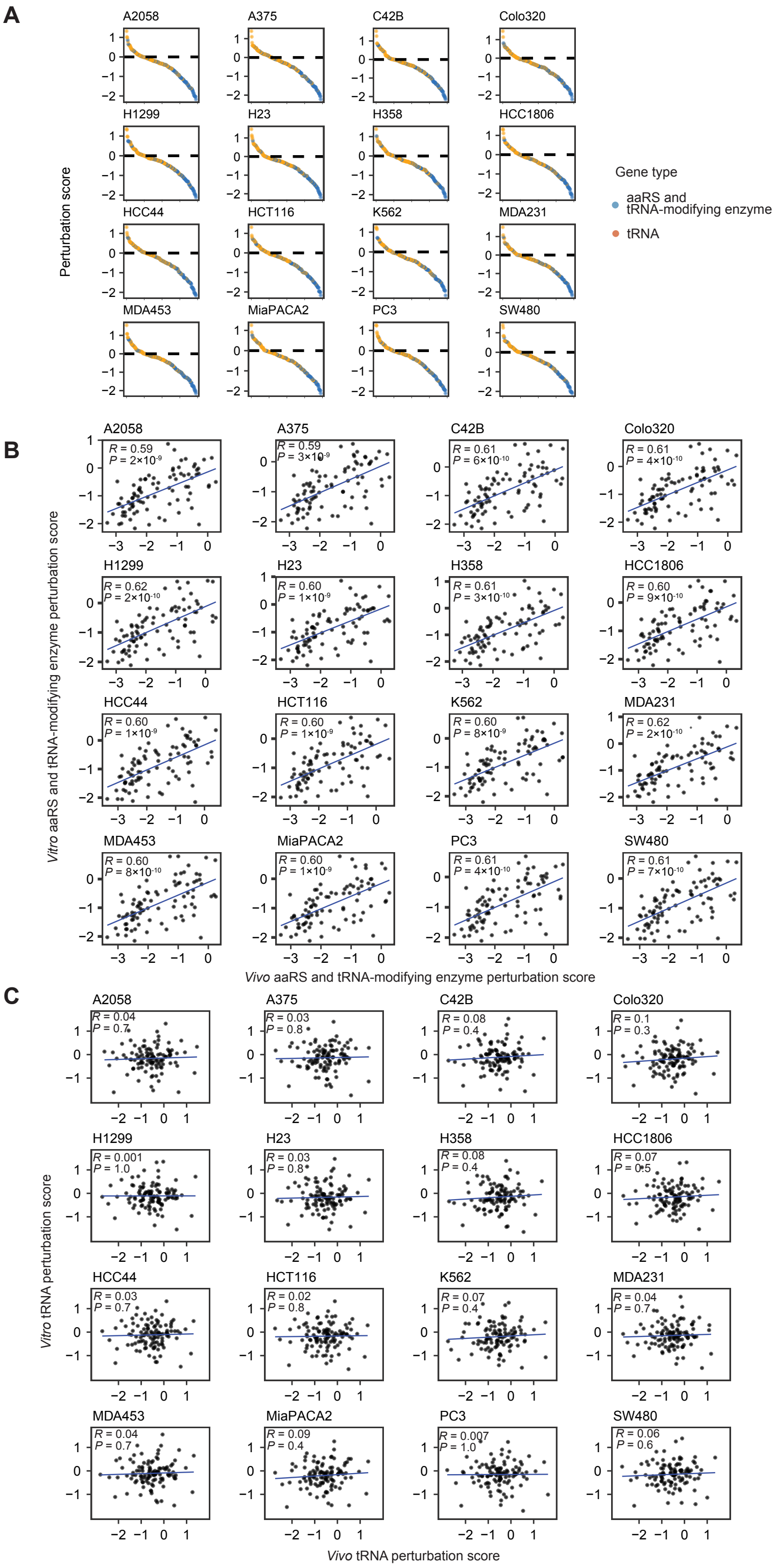

Figure S5 | Comparison of *in vivo* and *in vitro* CRISPRi perturbation effects. (A) Waterfall plots showing the distribution of perturbation effects for tRNA molecules, aminoacyl-tRNA synthetases (aaRSs), and tRNA-modifying enzymes across individual cell lines. **(B)** Scatter plot comparing the perturbation effects of aaRSs and tRNA-modifying enzymes between *in vivo* and *in vitro* CRISPRi experiments. Pearson correlation coefficients (R) and corresponding P values are shown. **(C)** Scatter plot comparing tRNA perturbation effects between *in vivo* and *in vitro* CRISPRi experiments. Pearson correlation coefficients (R) and corresponding P values are shown.

bioRxiv preprint doi: https://doi.org/10.64898/2026.10.08.757597; this version posted October 9, 2026. The copyright holder for this preprint (which was not certified by peer review) is the author/funder, who has granted bioRxiv a license to display the preprint in perpetuity. It is made available under a CC-BY 4.0 International license.

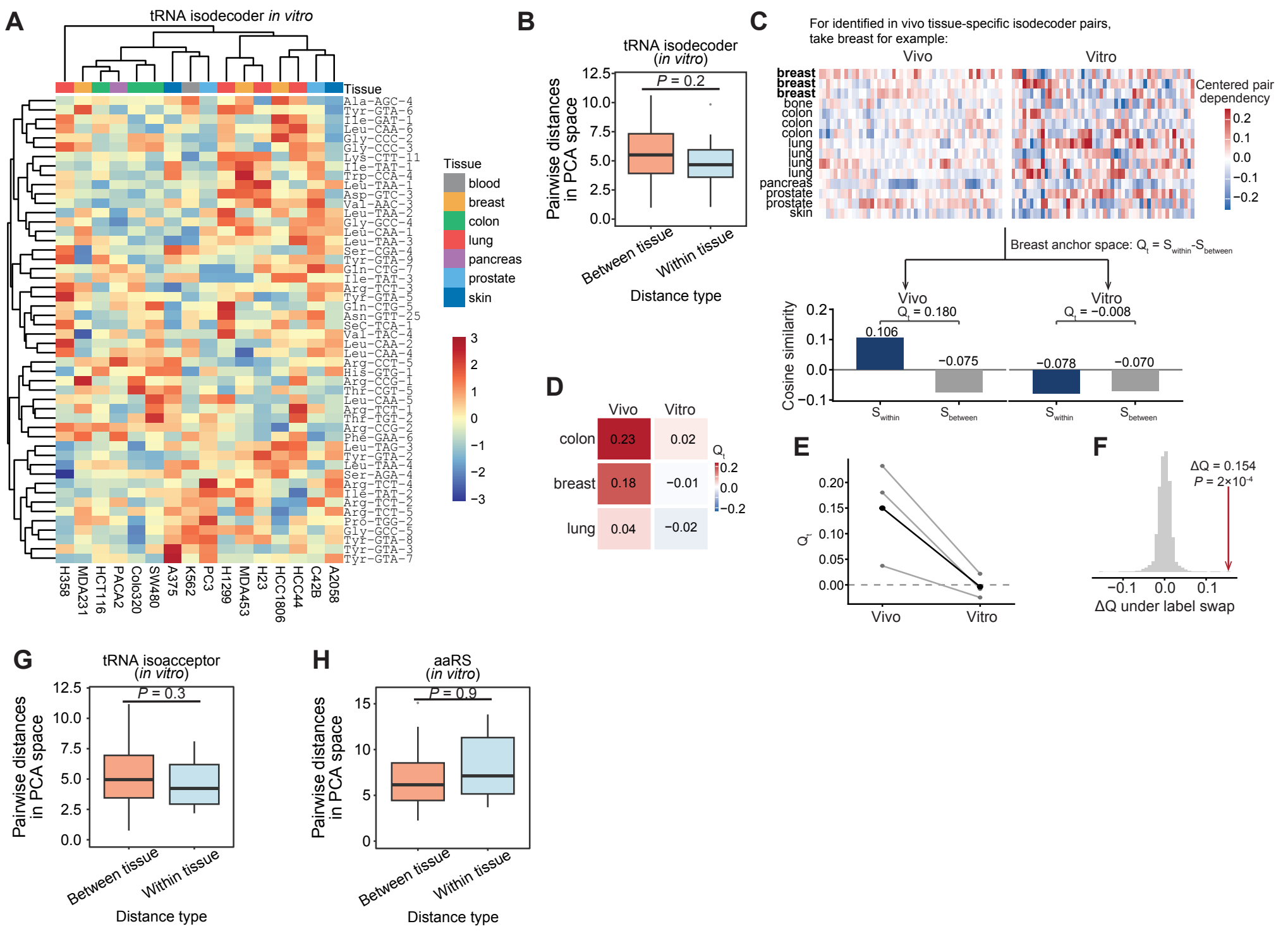

Figure S6 | Organization of tRNA dependencies *in vitro*. **(A)** Heatmap of *in vitro* tRNA isodecoder CRISPRi perturbation profiles across cell lines. Isodecoders and cell lines were hierarchically clustered using Spearman-correlation-based distances to group similar dependency patterns. **(B)** Pairwise Euclidean distances between cell lines in the PCA space shown in (**Fig. 3C**). Within-tissue distances were calculated between cell lines from the same tissue, whereas between-tissue distances were calculated between cell lines from different tissues. Within-tissue comparisons were restricted to tissues represented by at least three cell lines. Boxplots show the median, interquartile range, and 1.5× interquartile range; points indicate outliers. **(C)** Schematic and representative calculation of tissue-specific coherence using breast as the anchor tissue. Tissue-specific isodecoder pairs were identified from the *in vivo* dependency data (**Fig. S3**), and the same fixed pair set was evaluated under both *in vivo* and *in vitro* conditions. All 50 breast-specific isodecoder pairs are shown. **(D)** Tissue-specific coherence (Q_t) for each anchor tissue under *in vivo* and *in vitro* conditions. Tissues are ordered by the magnitude of the *in vivo*-to-*in vitro* change in coherence. All three tissues showed reduced coherence *in vitro*, with values approaching zero. **(E)** Paired comparison of tissue-specific coherence between *in vivo* and *in vitro* conditions. Gray lines denote individual anchor tissues, and the black line denotes the equally weighted mean across tissues. **(F)** Permutation test for the observed change in tissue-specific coherence. *In vivo* and *in vitro* condition labels were swapped at the cell-line level while retaining the tissue-specific pair sets, and pooled ΔQ was recalculated for each permutation. The inset shows the resulting null distribution, with the observed ΔQ indicated in red. **(G)** Pairwise within-tissue and between-tissue Euclidean distances in the *in vitro* isoacceptor PCA space, calculated as in **(B)**. **(H)** Pairwise within-tissue and between-tissue Euclidean distances in the *in vitro* aaRS dependency PCA space, calculated as in **(B)**.

bioRxiv preprint doi: https://doi.org/10.64898/2026.10.08.757597; this version posted October 9, 2026. The copyright holder for this preprint (which was not certified by peer review) is the author/funder, who has granted bioRxiv a license to display the preprint in perpetuity. It is made available under a CC-BY 4.0 International license.

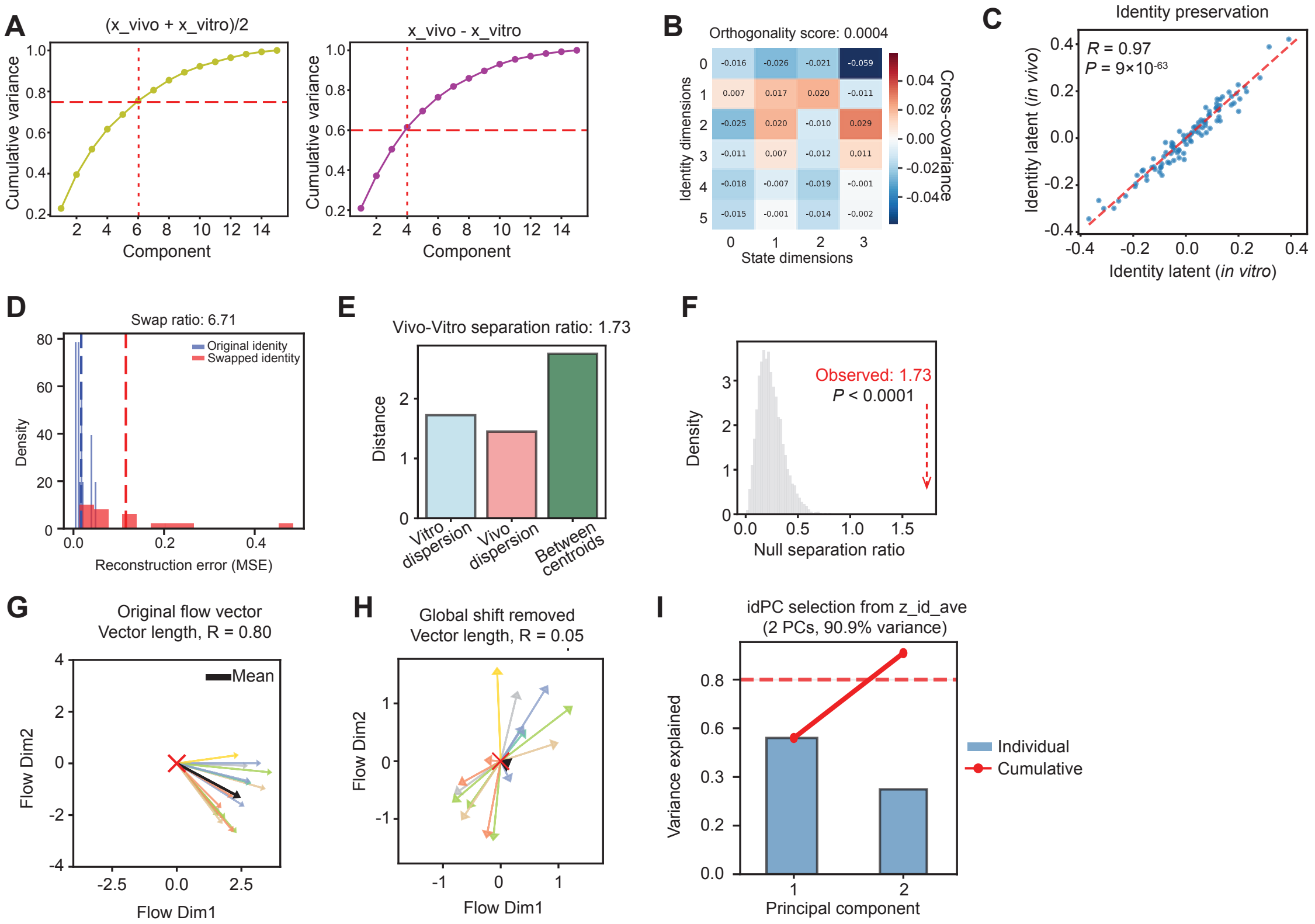

Figure S7 | Quality control of the pcVAE model. **(A)** PCA variance explained for vivo – vitro, and (vivo + vitro)/2, used to guide the determination of the number of latent dimensions representing state and identity in the pcVAE model. **(B)** Heatmap showing the cross-covariance between identity latents and state latents. **(C)** Scatter plot comparing *in vivo* and *in vitro* identity components derived from the pcVAE model. Pearson correlation coefficients (R) and corresponding P values are shown. **(D)** Histogram showing the reconstruction error distribution for original versus swapped identity representations. **(E** Bar plot showing the dispersion of *in vivo* and *in vitro* state representations and the separation between their centroids, as presented in Figure 4C. **(F)** Histogram showing the null distribution of the *in vivo*–*in vitro* separation ratio shown in **(F)** and Figure 4C, generated from 10,000 random shuffles. **(G)** Vector-field representation of the directional flow from *in vitro* to *in vivo* states, with all vectors anchored at a common origin. Directional coherence was quantified by the vector-strength metric R, defined as the length of the mean normalized flow vector, where $R = 1$ indicates perfect directional alignment and $R = 0$ indicates no net directional coherence. **(H)** Flow field after removal of the global adaptation component, highlighting tissue- or cell-dependent residual dynamics. **(I)** Bar plot showing the variance explained by each principal component of $z_{\text{id\_ave}}$, with the red line indicating cumulative variance explained.

bioRxiv preprint doi: https://doi.org/10.64898/2026.10.08.757597; this version posted October 9, 2026. The copyright holder for this preprint (which was not certified by peer review) is the author/funder, who has granted bioRxiv a license to display the preprint in perpetuity. It is made available under a CC-BY 4.0 International license.

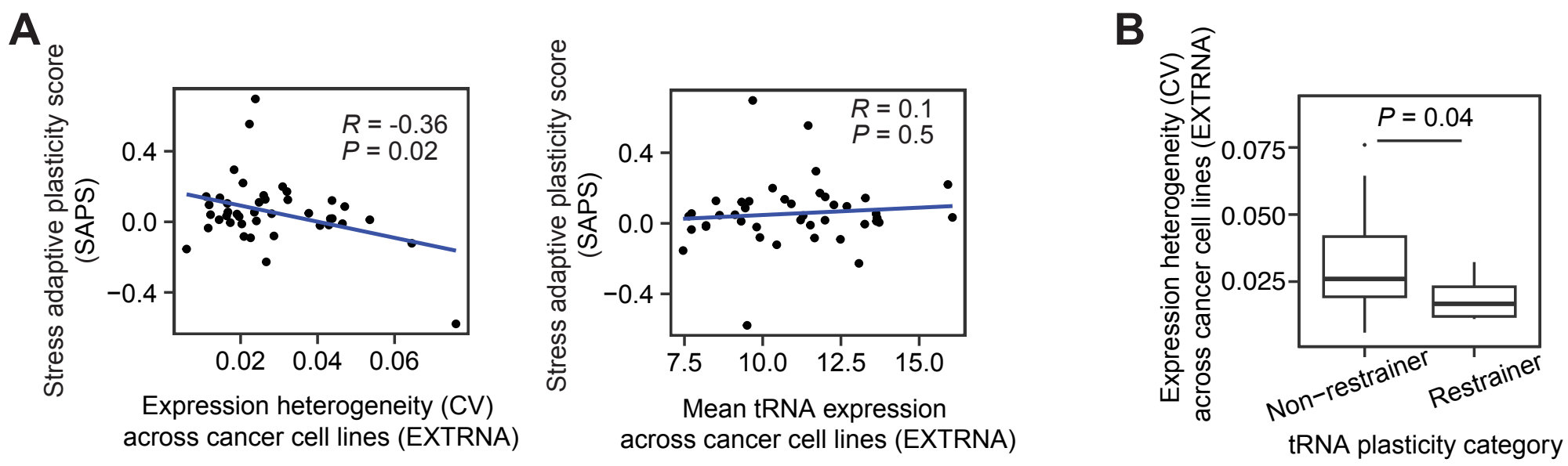

Figure S8 | Relationship between stress adaptive plasticity score and basal expression in standard culture in the EXTRNA dataset. (A) Scatter plots showing the association between the stress adaptive plasticity score and basal expression heterogeneity (CV) across cells (left) and mean expression levels (right) in the EXTRNA dataset. Pearson correlation coefficients (R) and corresponding P-values are shown. **(B)** Box plot showing the basal expression heterogeneity (CV) across cells for plasticity restrainer tRNAs compared with other tRNAs. P-value was calculated using Mann–Whitney U test.

bioRxiv preprint doi: https://doi.org/10.64898/2026.10.08.757597; this version posted October 9, 2026. The copyright holder for this preprint (which was not certified by peer review) is the author/funder, who has granted bioRxiv a license to display the preprint in perpetuity. It is made available under a CC-BY 4.0 International license.

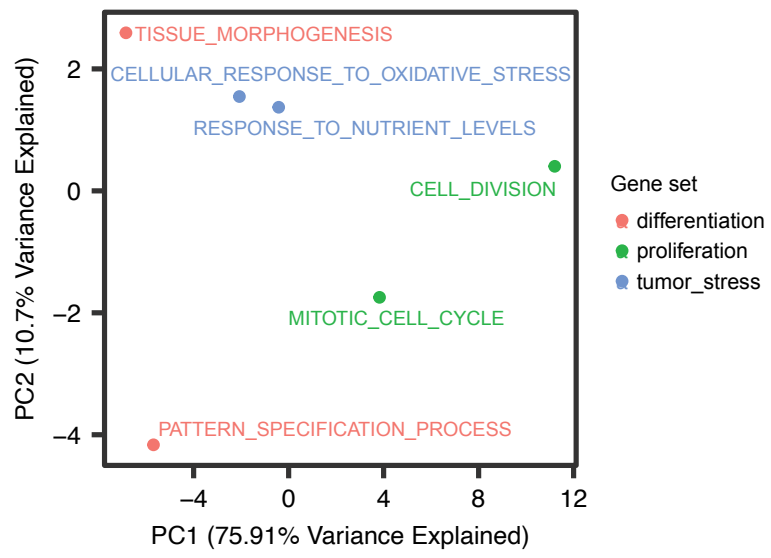

Figure S9 | Codon usage preferences associated with differentiation, proliferation, and stress response programs. Principal component analysis (PCA) showing codon usage preferences among genes associated with differentiation, proliferation, and stress response programs. Genes appearing in different GO term were excluded to ensure set-specific comparisons.

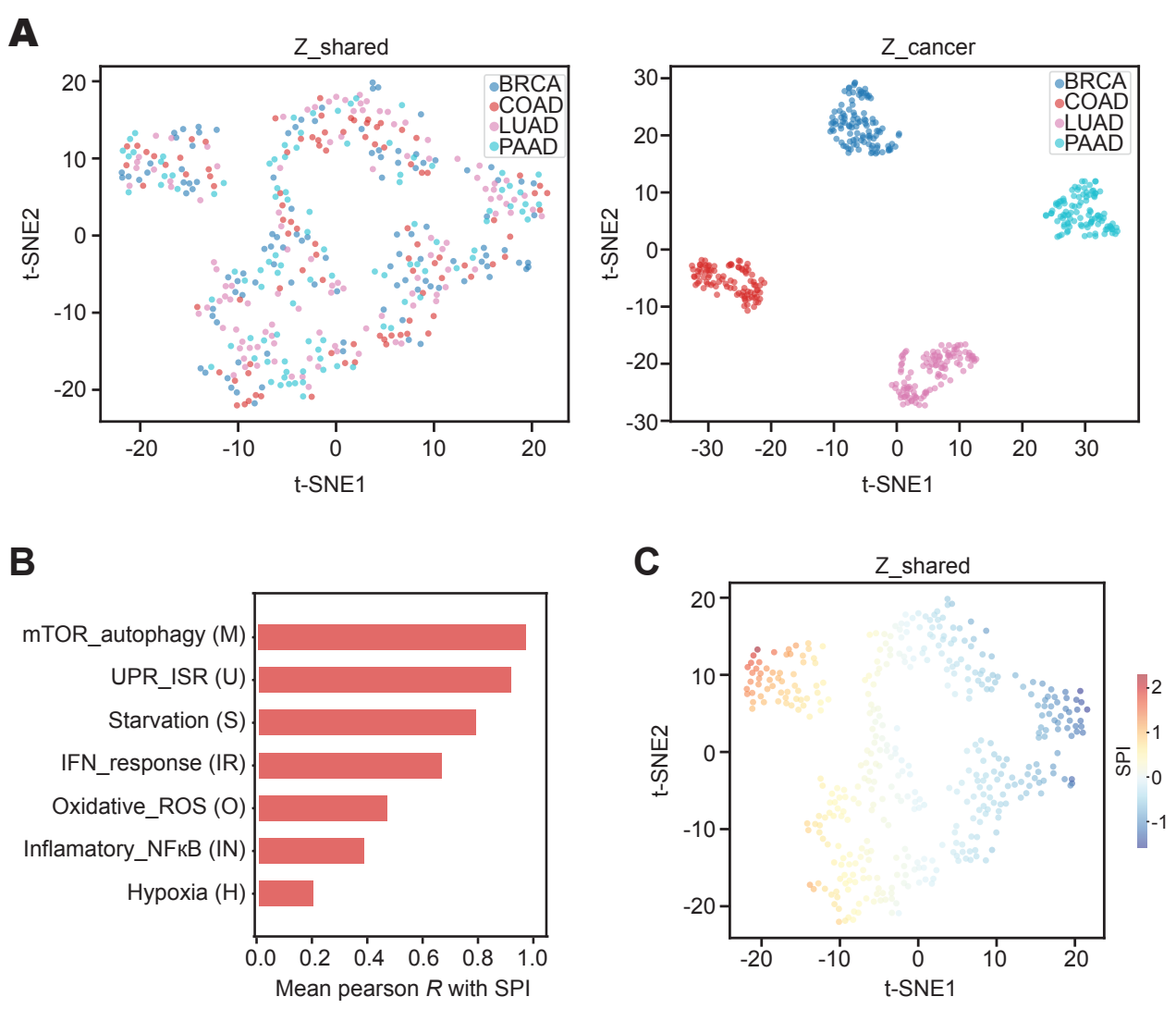

Figure S10 | Stress conditions across CPTAC. (A) t-SNE visualization of the conditional VAE model, showing separation of the shared latent space (z_{shared}, left) and the cancer-specific latent space (z_{cancer}, right) based on module scores of seven stress-associated terms. Points are colored by cancer type (BRCA, COAD, LUAD, and PAAD). **(B)** Bar plot showing the mean correlation coefficients between the calculated SPI and the module scores of each stress-associated term. **(C)** t-SNE visualization showing the SPI gradient in z_{shared}, with points colored by SPI values.

bioRxiv preprint doi: https://doi.org/10.64898/2026.10.08.757597; this version posted October 9, 2026. The copyright holder for this preprint (which was not certified by peer review) is the author/funder, who has granted bioRxiv a license to display the preprint in perpetuity. It is made available under a CC-BY 4.0 International license.

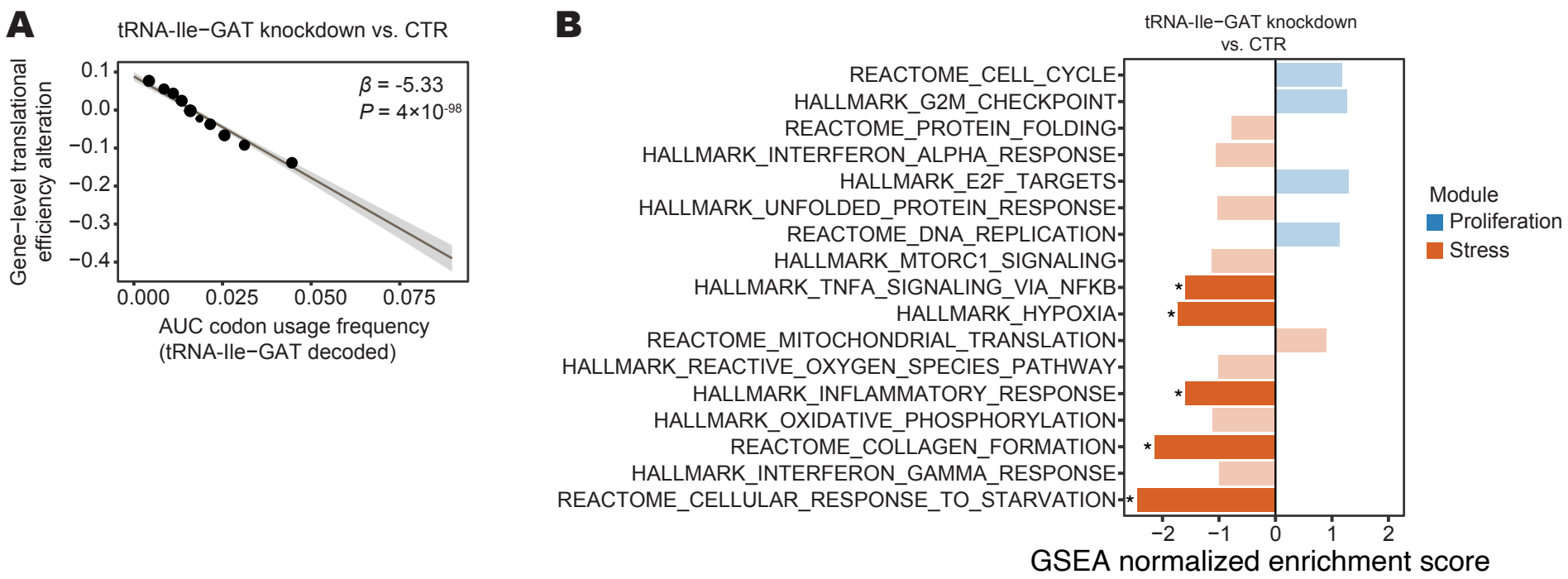

Figure S11 | tRNA-Ile-GAT depletion caused codon-dependent translational changes in tumor stress-associated programs. (A) Codon usage dependence of translational efficiency alterations following tRNA-Ile-GAT depletion. Differential translational efficiency between tRNA-Ile-GAT knockdown and control cells was estimated from matched RNA-seq and ribosome-profiling data. Points show genes grouped across the codon-usage distribution; the line and shaded region indicate the precision-weighted linear regression and 95% confidence interval, respectively. The regression coefficient (β) and P value are shown. **(B)** Pathway-level translational responses following tRNA-Ile-GAT depletion. Bars show normalized enrichment scores, with positive and negative values indicating enrichment toward increased and decreased translational efficiency, respectively. Asterisks indicate pathways with Benjamini–Hochberg-adjusted $P<0.05$
